# The Interplay of Uterine Health and Obesity: A Comprehensive Review

**DOI:** 10.3390/biomedicines12122801

**Published:** 2024-12-10

**Authors:** Dina Šišljagić, Senka Blažetić, Marija Heffer, Mihaela Vranješ Delać, Andrijana Muller

**Affiliations:** 1 Clinic of Gynecology and Obstetric, University Hospital Center Osijek, 31000 Osijek, Croatia; drdinasisljagic@yahoo.com (D.Š.); andrijana.muller@gmail.com (A.M.); 2 Department of Gynecology and Obstetrics, Faculty of Medicine, Josip Juraj Strossmayer University of Osijek, 31000 Osijek, Croatia; 3 Department of Biology, Josip Juraj Strossmayer University of Osijek, 31000 Osijek, Croatia; 4 Department of Medical Biology, School of Medicine, Josip Juraj Strossmayer University of Osijek, 31000 Osijek, Croatia; mheffer@mefos.hr; 5 Faculty of Veterinary Medicine, University of Zagreb, 10000 Zagreb, Croatia; mvranjes@vef.hr

**Keywords:** uterus, obesity, insulin resistance, leptin resistance, female reproductive health, metabolic syndrome, adipokines, endometrial function, fertility, therapeutic interventions

## Abstract

Uterine physiology encompasses the intricate processes governing the structure, function, and regulation of the uterus, a pivotal organ within the female reproductive system. The escalating prevalence of obesity has emerged as a significant global health issue, profoundly impacting various facets of well-being, including female reproductive health. These effects extend to uterine structure and function, influencing reproductive health outcomes in women. They encompass alterations in uterine morphology, disruptions in hormonal signaling, and inflammatory processes. Insulin and leptin, pivotal hormones regulating metabolism, energy balance, and reproductive function, play crucial roles in this context. Insulin chiefly governs glucose metabolism and storage, while leptin regulates appetite and energy expenditure. However, in obesity, resistance to both insulin and leptin can develop, impacting uterine function. Inflammation and oxidative stress further exacerbate the development of uterine dysfunction in obesity. Chronic low-grade inflammation and heightened oxidative stress, characteristic of obesity, contribute to metabolic disruptions and tissue damage, including within the uterus. Obesity significantly disrupts menstrual cycles, fertility, and pregnancy outcomes in women. The accumulation of excess adipose tissue disrupts hormonal equilibrium, disturbs ovarian function, and fosters metabolic irregularities, all of which detrimentally impact reproductive health.

## 1. Introduction

The uterus is a dynamic and complex organ central to female reproductive health. While its primary function is in reproduction, its roles extend beyond this, impacting overall health and disease states related to endocrine [1,2], immune [3,4,5], cardiovascular [6,7,8], neurological [9,10,11], and metabolic systems [12,13]. Understanding these roles is crucial for comprehensive women’s health care, influencing treatment strategies and improving quality of life. Further research into these non-reproductive roles will continue to illuminate the uterus’s full impact on overall health. Obesity, defined by an excessive accumulation of body fat, typically measured by a body mass index (BMI) of 30 or higher, is a chronic complex disease and one of the major factors impairing health. According to the WHO, in 2022, one in eight people in the world were living with obesity [14]. The rising prevalence of obesity poses significant challenges to female reproductive health. It impacts menstrual regularity, fertility, and pregnancy outcomes and increases the risk of reproductive cancers [15,16].

Pandey (2010) further emphasizes the association between obesity and gynecological issues, such as polycystic ovary syndrome and endometrial polyps [17]. Brown (2010) discusses the genetic contribution to obesity and its implications for reproduction. These studies collectively underscore the urgent need for interventions to address the growing burden of obesity on female reproductive health [18].

Insulin and leptin resistance are critical metabolic disturbances with profound implications for uterine function and female reproductive health. They contribute to a range of reproductive disorders, from menstrual irregularities and infertility to pregnancy complications [19,20] and increased cancer risk [21,22]. Addressing these resistances through lifestyle interventions, medical treatment, and ongoing research is essential for improving reproductive health outcomes. Understanding the mechanisms by which these resistances affect the uterus can help in developing targeted therapies and preventive strategies for women affected by these conditions.

The connection between obesity and uterine health is vital to understanding and managing various reproductive health issues. Overall, the research in this area can lead to better prevention, diagnosis, and treatment strategies, ultimately improving women’s health and quality of life.

## 2. Impact of Obesity on Uterine Reproductive and Immune Functions

### 2.1. Effects of Obesity on Uterine Structure

The uterus, positioned within the pelvic cavity between the bladder and rectum, is a hollow, muscular organ. Comprising three primary layers—the endometrium (inner lining), myometrium (middle muscular layer), and perimetrium (outer layer)—it undergoes shape variations throughout a woman’s life. These changes manifest during puberty, menstruation, pregnancy, and menopause [23,24]. Obesity affects uterine structure and function through a combination of hormonal imbalances [25], chronic inflammation [26], and metabolic disturbances [27]. These effects can lead to a range of reproductive health issues, including endometrial hyperplasia, irregular menstrual cycles [28], increased risk of uterine fibroids [29], endometrial cancer [30], and pregnancy complications [31].

The relationship between estrogen and obesity is bidirectional. Increased adipose tissue in obesity leads to higher levels of circulating estrogen synthesized and metabolized by the cytochrome P450 (CYP) superfamily of enzymes, specifically aromatase CYP19A1. This prolonged estrogen exposure, without the balancing effect of progesterone, can cause the endometrial lining to thicken excessively, resulting in endometrial hyperplasia [24,32]. This condition can lead to abnormal uterine bleeding and increase the risk of endometrial cancer [33]. Deficiency of estrogen leads to excessive fat accumulation and impairs adipocyte function; on the other hand, adipose tissue of obese individuals is characterized by altered expression of estrogen receptors and key enzymes involved in their synthesis [34]. Additionally, obesity is associated with increased androgen levels, likely mediated by insulin resistance, which may also have implications for uterine health [35]. This relationship will be further explored in Section 3.

Obesity-related hormonal imbalances, particularly elevated estrogen levels, are thought to contribute to the development and growth of uterine fibroids (uterine leiomyomas). These are benign tumor changes that originate from the smooth muscle layer of the uterus. Leiomyoma growth is a consequence of hormonal action that acts through their estrogen and progesterone receptors [36,37]. Fibroids can affect uterine structure and function by causing abnormal uterine bleeding, pelvic pain, and infertility, depending on their size and location [29].

### 2.2. Obesity and Uterine Reproductive Function

The uterus plays a crucial role in the menstrual cycle, as it is central to the preparation for a possible pregnancy each month. A complex interplay of hormones, mainly estrogen and progesterone, produced by the ovaries, governs the menstrual cycle in all phases—menstrual, follicular, ovulatory, and luteal. The uterus prepares for potential pregnancy by thickening the endometrial lining each month during the proliferative phase of the menstrual (uterine) cycle in response to hormonal changes, mainly estrogen and progesterone. If fertilization does not occur, the endometrial lining sheds, leading to menstrual bleeding. The uterus contracts to expel this lining, which can cause cramping. Chronic exposure to elevated levels of estrogen due to increased adipose tissue can lead to endometrial hyperplasia, characterized by abnormal thickening of the endometrial lining [38,39,40]. Endometrial hyperplasia is a risk factor for endometrial cancer, particularly in postmenopausal women [41,42]. Conditions such as polycystic ovary syndrome (PCOS), characterized by irregular menstrual cycles, ovarian dysfunction, and hyperandrogenism, are more prevalent in women with obesity and can further impair fertility [43,44,45]. Also, evidence shows that excessive weight can negatively impact reproductive health by altering endometrial gene expression and reducing endometrial receptivity [46]. The uterus must be free of abnormalities, like scarring or fibroids, to support a full-term pregnancy. Obese women more frequently experience prolonged pregnancies, and they have a higher chance of needing interventions during labor and delivery, such as cesarean section, due to factors such as macrosomia, labor dystocia, and inadequate uterine contractions [47,48,49,50]. Studies on uterine contractility in obese women have shown mixed results. Some studies indicated differences in contraction of myometrial strips from obese women, who contracted less frequently and with less force than those from nonobese women [51], while other studies reported no differences in contraction strength and frequency [52,53]. Also, the typically higher cholesterol levels in obese women alter myometrial myocyte cell membranes, particularly the caveolae, which inhibits oxytocin receptor function and increases K+ channel activity, resulting in preventing the uterus from contracting [54], while dysfunctional labor patterns and increased oxytocin use in obese women may not be attributed to differences in oxytocin expression [55]. A high-fat diet accumulates big fat droplets in the stromal layers of the uterus, ovary, and oviducts, with the consequence of altering their histological integrity, and in women, changes like hyperproliferative uterus, vacuolated ovarian tissue, and thickened oviduct walls occur [56]. Maternal obesity in humans and in high-fat diet (HFD) mice is associated with impaired uterine vascular remodeling at the maternal side of the placental–uterine interface, characterized by the presence of smooth muscle layers around arteries and narrower arterial conduits [57,58,59,60].

### 2.3. Obesity and Uterine Immune Function

Inflammation and oxidative stress play significant roles in uterine dysfunction associated with obesity by affecting endometrial health, reducing fertility, and increasing the risk of pregnancy complications [61]. Obesity is characterized by chronic low-grade inflammation, marked by elevated levels of inflammatory cytokines like TNF-α, IL-6, and CRP [62]. Systemic inflammation in obesity can also affect the uterus through circulation, as pro-inflammatory cytokines can cross the blood-uterine barrier and exert direct effects on uterine tissue [63]. Inflammation alters the gene expression and cellular signaling within the endometrium, impairing its ability to prepare for and support implantation [64]. Chronic inflammation may also interfere with endometrial receptivity, crucial for successful embryo implantation. Besides that, it is also linked to negatively impacting egg quality and embryo implantation in the uterus, raising the risk of miscarriage [65]. Pro-inflammatory cytokines can negatively alter the expression of adhesion molecules and cytokines involved in embryo implantation, potentially leading to implantation failure and infertility [66]. Chronic inflammation in the endometrium may also contribute to the development of endometrial disorders such as endometriosis and endometrial hyperplasia, which are associated with infertility and an increased risk of endometrial cancer [67]. Even excessive inflammation can be detrimental; insufficient immune activity may also impair uterine function and embryo invasion, underscoring the need for a delicate balance [68]. A controlled immune response is critical for processes such as endometrial remodeling during the menstrual cycle (especially during the proliferative phase of the uterine cycle) and supporting embryo implantation [69]. Uterine natural killer (uNK) cells in the endometrium have a crucial role in implantation and healthy pregnancy by producing cytokines and growth factors that promote uterine blood vessel growth and support embryo development [70]. Oxidative stress in the uterus can manipulate the immune system by both activating the inflammatory pathways and causing the immune cells, like macrophages, T cells, and natural killer (NK) cells, to change their behavior [61,71]. Obesity often leads to an imbalance between the production of ROS and the body’s antioxidant defenses, resulting in oxidative stress. High levels of ROS can damage endometrial cells and impair uterine function [72]. Oxidative stress can disrupt the delicate processes of ovulation, implantation, and early embryo development by damaging the endometrial lining and cellular components, including lipids, proteins, and DNA, leading to cellular dysfunction and tissue damage in the uterus by inhibition of myometrial contraction, which contributes to dysfunctional labor [72,73]. Recent studies indicate a possible association between obesity and the endometrial microbiome [74], which is also part of the immune response. In obese mothers, myometrial arteries exhibit deficits in endothelial cell calcium signaling, and the eNOS system is modified to both contractile and relaxation responses [75].

## 3. Insulin Resistance and Uterine Health

### 3.1. Insulin Signaling in the Uterus

Insulin signaling within the uterus is one of the major factors controlling cellular metabolism and development, as well as the processes of cellular growth. Insulin and insulin-like growth factors 1 and 2 (IGF1 and IGF2) bind to their own receptors, resulting in the activation of two important pathways, PI3K-Akt and Ras-MAPK, important in regulating gene expression and the regulation of epithelial cell proliferation [76]. Insulin receptors are expressed in the endometrium, and aberrant insulin signaling may disrupt cellular proliferation, differentiation, and apoptosis processes [77]. Dysregulated insulin signaling may lead to abnormal endometrial growth and remodeling, contributing to conditions such as endometrial hyperplasia and dysfunctional uterine bleeding [78,79]. Endometrial proliferation and implantation in mice is regulated by insulin signaling, and insulin receptor and IGF1R are important for embryo implantation, while uterine receptivity is unaffected by insulin receptor ablation [76].

### 3.2. Mechanisms Linking Insulin Resistance and Uterine Dysfunction

Insulin resistance contributes to uterine dysfunction through several mechanisms: hormonal imbalance (increased androgens), direct impairment of endometrial cell function, chronic inflammation, oxidative stress, and altered progesterone signaling. These factors together disrupt the normal uterine environment, leading to menstrual irregularities, impaired fertility, and an increased risk of pregnancy complications. Insulin resistance can directly affect endometrial function by altering insulin signaling pathways in uterine tissue. The processes of angiogenesis and endometrial repair are not only strongly estrogen- and progesterone-dependent but also involve NO, platelets, immune cells, and numerous growth factors [80,81]. IR occurs in decrease of NO production, which leads to increase in the shear stress on endothelial cells because of diffuse vasoconstriction. Mutual action of IR, decreased NO and hyperglycemia generate the release of inflammatory cytokines and free radical formation, which leads to endothelial damage, surrounding tissue hypoxia, and possible cellular apoptosis [82]. Elevated insulin levels in vivo modulate apoptosis via the PI3K-Akt pathway, decrease receptors for bovine serum albumin 2 (BMP2), a key decidual marker, impair estrogen and progesterone signaling, further damage endometrial stromal cells, and enhance mitochondrial transmembrane potential, thereby disrupting endometrial decidualization [83]. Resistance-induced hyperinsulinemia can also stimulate the production of insulin-like growth factor 1 (IGF-1) [84], which may promote endometrial cell proliferation and increase the risk of endometrial cancer. Chronic inflammation alters the expression of genes involved in uterine receptivity and cellular function [85], further reducing the likelihood of successful implantation and increasing the risk of uterine dysfunction. Mechanisms of oxidative stress affect endometrial cell health, causing DNA damage, cell death, and an impaired response to hormonal signals [86], all of which are crucial for a functional and receptive endometrium. Inflammatory mediators, such as TNF-α and IL-6, can disrupt normal endometrial function, impair embryo implantation, and increase the risk of pregnancy complications [87]. The vascular changes allow different factors to leave the blood and form plaques in the blood vessel tunica intima layer. This thickening of the blood vessels leads to increased vascular resistance, together with chronic low-grade inflammation, and leads to atherosclerosis [82,88,89]. Insulin resistance can also affect how the uterus responds to progesterone, the hormone critical for maintaining a healthy endometrial lining during the second half of the menstrual cycle [90]. This can disrupt the menstrual cycle and lead to issues like endometrial hyperplasia or irregular shedding of the uterine lining. A common feature of obesity and conditions like PCOS is closely linked to uterine dysfunction [91]. Women with PCOS often experience insulin resistance, which is strongly linked to anovulation and thickened, dysfunctional endometrial lining, leading to fertility problems. Insulin-mediated glucose transporter factor-4 (GLUT4) facilitates glucose transport to endometrial glandular tissue. Fornes et al. demonstrated reduced levels of GLUT4, insulin pathway proteins, and their phosphorylation in the endometrial tissues of PCOS patients, indicating the presence of localized insulin resistance in the endometrium of these patients [92]. Reduced glucose transport function would cause glucose deficiency in the endometrial cells, which would affect the normal growth of the endometrial cells and lead to embryo implantation failure or increased risk of miscarriage.

Impaired endometrial receptivity in patients with PCOS in humans may be the reason endometrial stromal cells and excess TNF-α negatively affect insulin sensitivity and lead to abnormal energy metabolism by decreasing lipocalin signaling and blocking GLUT-4 membrane transport [93].

### 3.3. Role of Hyperinsulinemia in Uterine Pathology

In insulin resistance, the body compensates by producing more insulin, creating the state of hyperinsulinemia. Hyperinsulinemia plays a central role in the development of endometrial pathology and menstrual irregularities through its effects on estrogen production, androgen levels, and insulin-like growth factors. Elevated insulin levels stimulate the ovaries to produce excess androgens (male hormones like testosterone), which can disrupt the normal hormonal balance needed for healthy reproductive function. High androgen levels can alter the endometrial environment, leading to irregular menstrual cycles, poor endometrial receptivity, and difficulty with embryo implantation [94], which precedes the process of decidualization dependent on the steroid hormone progesterone acting through the nuclear progesterone receptor (PR) that works through insulin receptor substrate 2 (IRS2) expression [90]. Insulin acts as a growth factor in the endometrium, stimulating the proliferation of endometrial cells [95]. Hyperinsulinemia, a common feature of conditions such as PCOS and hyperinsulinemia type A, has been linked to endometrial dysfunction (hyperplasia [96] and endometrial cancer), menstrual irregularities [92,93], and infertility [97]. These findings suggest a potential role of hyperinsulinemia in the pathophysiology of endometrial pathology and menstrual irregularities. Insulin influences estrogen metabolism by increasing ovarian androgen production and decreasing the liver’s production of sex hormone-binding globulin (SHBG) [98]. This results in higher circulating levels of free estrogen. Since estrogen stimulates the growth of the endometrial lining, chronic exposure to high estrogen levels without the counterbalance of progesterone (which normally happens in anovulatory cycles) can lead to endometrial thickening and hyperplasia. In addition to influencing estrogen, insulin itself acts as a growth-promoting hormone. In the endometrium, this promotes cellular growth and increases the risk of abnormal tissue development [99]. Combined with estrogen’s effects, hyperinsulinemia accelerates endometrial growth, increasing the risk of abnormal pathology, including hyperplasia and possibly even endometrial cancer.

### 3.4. Pregnancy Complications and Fertility Treatments in Insulin-Resistant Individuals

Insulin resistance significantly impacts fertility and pregnancy outcomes. In individuals with IR, metabolic, hormonal, and inflammatory disturbances can affect ovulation, endometrial receptivity, and fetal development, leading to challenges in fertility treatments and increased pregnancy complications. Wells (1960) noted that insulin administration in pregnant diabetic rats improved fertility and maternal mortality, although fetal mortality remained higher [100]. Seely (2003) suggested that insulin resistance may play a role in pregnancy-induced hypertension, highlighting the need for interventions to reduce insulin resistance and potentially mitigate these risks [101]. Women with IR may require ovulation induction drugs like clomiphene citrate or letrozole to stimulate ovulation [102]. However, insulin resistance can make these treatments less effective, often requiring higher doses or additional medications to achieve successful ovulation. Medications like metformin (an insulin-sensitizer) are frequently used in fertility treatments for women with IR. Metformin helps reduce insulin levels, improve ovulation, and enhance the effectiveness of ovulation induction therapies. In women with PCOS, metformin is often prescribed to regulate cycles and improve pregnancy rates [103,104,105]. Despite ovulation induction, many insulin-resistant individuals experience implantation failure due to the disrupted uterine environment [106]. Insulin-sensitization treatments can improve outcomes, but this remains a significant challenge for fertility treatments. Insulin resistance and associated metabolic disturbances may impact the success rates of IVF treatments. Women with insulin resistance may have lower ovarian reserve, reduced oocyte quality, and decreased embryo implantation rates compared to non-insulin-resistant individuals. However, addressing insulin resistance prior to IVF may improve outcomes [107]. During pregnancy, insulin resistance increases the risk of complications such as miscarriage, gestational diabetes, pre-eclampsia, fetal growth abnormalities, and preterm birth. Malaza et al. reported more adverse pregnancy outcomes in women with pregestational diabetes compared to those with gestational diabetes. These complications include cesarean section, preterm birth, congenital anomalies, pre-eclampsia, neonatal hypoglycemia, macrosomia, neonatal intensive care unit admission, stillbirth, low Apgar score, large for gestational age infants, induction of labor, respiratory distress syndrome, and miscarriages [108]. This aligns with findings from the International Prospective Hyperglycemia and Adverse Pregnancy Outcomes (HAPO) study group [109]. Insulin, as an anabolic hormone, plays a key role in regulating fetal growth. Maternal hyperglycemia leads to fetal hyperglycemia and hyperinsulinemia, which stimulates anabolism and the development of muscle, adipose, and connective tissue. These processes contribute to increased storage of fetal fat and protein, resulting in macrosomia [110]. Careful management and monitoring throughout fertility treatments and pregnancy are essential to optimize outcomes for both the mother and baby. Standard therapy for gestational diabetes requiring insulin therapy [111,112] has shown better maternal and newborn outcomes compared to diet, oral anti-diabetic drugs, or insulin analogues, with a lower incidence of macrosomia [113]. In the management of diabetes during pregnancy, metformin monotherapy is effective in maintaining glycemic control [114] and is specifically recommended for patients with prediabetes or a BMI of 35 kg/m^2^ [115].

## 4. Leptin Resistance and Uterine Health

Leptin resistance is a condition in which the body becomes less responsive to the hormone leptin, despite having elevated leptin levels. Leptin, primarily produced by fat cells, plays a key role in regulating energy balance, appetite, and reproductive functions. In the context of uterine function, leptin resistance has significant implications for reproductive health, particularly in individuals with obesity or metabolic disorders.

### 4.1. Leptin Signaling in the Uterus

Leptin, a hormone secreted by adipose (fat) tissue, serves as a key regulator of energy balance and reproduction, specifically by preparing the endometrium for implantation by influencing factors like angiogenesis (formation of new blood vessels) and cytokine regulation [116]. Leptin affects the uterus through the leptin receptors (Ob-R), belonging to the class I cytokine receptor family, that are expressed in the uterine epithelium, stroma, myometrium, and endometrial glands [117]. The presence of leptin receptors suggests that the uterus is responsive to leptin signaling, which can modulate various cellular processes involved in uterine function. When leptin attaches to Ob-R, it activates the JAK-STAT signaling pathway that promotes gene expression related to growth, differentiation, and inflammatory processes required for the maintenance of the uterine lining and for the embedding of the embryo [118]. Leptin signaling is essential for coordinating energy balance with reproductive function, particularly in the uterus. It regulates the menstrual cycle, enhances endometrial receptivity, supports implantation, and ensures proper pregnancy maintenance. Leptin signaling can also activate the MAPK pathway, influencing cell growth and survival in the uterine tissues. This pathway is important for controlling the structural integrity of the endometrium during the menstrual cycle and early pregnancy [119]. Another significant pathway affected by leptin is the phosphatidylinositol-3-kinase PI3K-Akt pathway, which is involved in cellular metabolism and survival [120] by regulating the metabolic demands of uterine cells and ensuring proper nutrient supply during pregnancy. Both mentioned pathways regulate the promotion of endometrial growth and regeneration [21]. Leptin resistance reduces the ability of the endometrium to respond to these signals, potentially leading to implantation failure and infertility [121]. Understanding and managing leptin signaling pathways is crucial for improving reproductive health and addressing fertility issues linked to metabolic imbalances.

### 4.2. Disruption of Leptin Signaling in Obesity and Uterine Health

In a healthy physiological state, leptin plays an essential role in regulating reproductive processes like regulation of the menstrual cycle and endometrial receptivity. Different studies indicated significant variations in serum leptin during the female menstrual cycle, but results are not uniform. Many previous studies have shown conflicting results related to the leptin levels during the menstrual cycle. Some researchers noted that the leptin concentration increases gradually from the first phase, reaching the highest concentration in the luteal phase [122,123], while others noted a highest concentration in the pre-ovulatory phase [124]. The disruption in leptin signaling, particularly leptin resistance seen in metabolic disorders like obesity, has profound consequences for uterine health and overall reproductive function, leading to significant reproductive dysfunctions, including infertility, poor pregnancy outcomes, and uterine pathologies [125]. Leptin is involved in the regulation of the hypothalamic–pituitary–gonadal (HPG) axis, influencing the secretion of hormones such as gonadotropin-releasing hormone (GnRH), luteinizing hormone (LH), and follicle-stimulating hormone (FSH). These hormones are critical for ovulation and the maintenance of a regular menstrual cycle. It also modulates the immune environment in the uterus to support early pregnancy [126]. Leptin resistance reduces the ability of leptin receptors (Ob-R) in the endometrium to respond to the hormone [116,127]. This impairs the regulatory function of leptin on cellular proliferation, immune defense, and the development of blood vessels within the uterine cavity structure, leading to a situation where the endometrium does not adequately react to hormonal stimuli towards its readiness for embryo attachment [64]. When this signaling is impaired, the uterine lining is less capable of supporting the attachment and growth of an embryo, leading to implantation failure and reduced fertility [128]. Leptin promotes the development of new blood vessels in the endometrium through its regulation of vascular endothelial growth factor (VEGF) [129]. In obesity, disrupted leptin signaling compromises this process, leading to poor uterine vascularization and a suboptimal environment for embryo implantation [130,131]. The impaired leptin signaling in obesity disrupts normal ovarian function and further contributes to infertility in PCOS patients [132]. Leptin resistance reduces the uterus’s ability to support early pregnancy by compromising endometrial receptivity and placental development [133]. As a result, women with obesity and leptin resistance face a higher risk of early pregnancy loss. Addressing leptin resistance through weight management, lifestyle changes, and pharmacological interventions can help improve reproductive health and enhance fertility in individuals with obesity.

### 4.3. Potential Therapeutic Targets to Restore Leptin Sensitivity in the Context of Obesity

Restoring leptin sensitivity in obesity is a complex challenge, as leptin resistance often involves multiple pathways and factors. Correia (2007) and Santoro (2015) both highlight the potential of restoring leptin sensitivity as a treatment target, with Santoro emphasizing the need for new therapeutic tools [134,135]. GLP-1 receptors are widely distributed throughout the reproductive system, and both preclinical models and some clinical studies suggest that GLP-1 plays a key role in linking the reproductive and metabolic systems. GLP-1 also appears to have anti-inflammatory and anti-fibrotic effects in the gonads and endometrium, particularly in conditions like obesity, diabetes, and polycystic ovary syndrome (PCOS) [136]. Sáinz (2015) suggests that anti-inflammatory drugs or bioactive food components could help overcome leptin resistance [137], while Salazar (2019) underscores the importance of addressing leptin resistance in the context of type 2 diabetes [138]. These studies collectively underscore the need for further research to identify and develop effective therapeutic targets for restoring leptin sensitivity in obesity. Based on the in vitro studies, there are some potential therapeutic targets and strategies. Enhancing leptin signaling through the JAK2/STAT3 pathway can potentially restore sensitivity. This involves targeting molecules that modulate the Janus kinase 2 (JAK2) and signal transducer and activator of transcription 3 (STAT3) proteins [139]. Also, suppressing Suppressor of Cytokine Signaling (SOCS) proteins, especially SOCS3, which inhibit leptin signaling, could help restore leptin sensitivity [140]. Targeting inflammatory cytokines (e.g., TNF-α, IL-6) or signaling pathways (e.g., NF-κB) could reduce leptin resistance [141]. Improving insulin sensitivity can often help with leptin resistance. The gut microbiota plays a role in systemic inflammation and metabolic processes. Probiotics or prebiotics might help in restoring leptin sensitivity by influencing gut health [142]. Certain dietary components and bioactive compounds have been shown to modulate leptin signaling and improve leptin sensitivity. Omega-3 fatty acids, found in fish oil and certain plant sources, have anti-inflammatory properties and may enhance leptin signaling [143]. Polyphenols, such as resveratrol found in red grapes and berries, have been shown to enhance insulin sensitivity and modulate leptin signaling pathways. They achieve this by suppressing leptin expression in adipocytes, reducing hyperleptinemia, and alleviating leptin resistance [144]. Pharmacological leptin sensitizers include small molecules that produce modest weight loss when used alone but significantly amplify the anorectic effects of exogenous leptin. Examples include meta-chlorophenylpiperazine, a serotonin receptor agonist and reuptake inhibitor; metformin, a widely used anti-diabetic medication; and betulinic acid, which likely enhances leptin sensitivity through PTP1B inhibition [145,146,147]. Each of these strategies has its own set of challenges and potential benefits. Along with dietary manipulations to restore leptin sensitivity, exercise also decreases body weight and leptinemia and improves leptin sensitivity by activating leptin sensitive neurons in the VMH and increasing pSTAT3 in the VTA. This effect seems to be independent of fat mass loss [148,149]. Research is ongoing to better understand how these various factors interact and how best to target them therapeutically.

## 5. Future Directions and Conclusions

The relationship between uterine health and obesity is complex and deeply intertwined. Obesity influences uterine function through metabolic, hormonal, and inflammatory pathways. Obesity leads to insulin resistance and an imbalance in circulating adipokines, characterized by increased levels of leptin and decreased levels of adiponectin. Such changes can disrupt the normal physiological function of the uterus. In addition, such changes create an environment for chronic inflammation that has been associated with several conditions of the uterus, including endometrial hyperplasia, fibroids, and polycystic ovary syndrome (PCOS). Additionally, obesity-induced hormonal imbalances, such as elevated estrogen levels, can affect the uterine lining, increasing the risk of conditions like endometrial cancer.

The impact of obesity on uterine health is further compounded by alterations in the uterine microbiome and epigenetic modifications that may influence long-term uterine function and fertility. Obesity also affects uterine blood flow, contributing to pregnancy complications such as preeclampsia. These factors together contribute not only to poor fertility and pregnancy outcomes but also to the enhanced risk of reproductive disorders and cancers.

This would be beneficial in targeted therapies and prevention strategies to improve uterine health among obese individuals. As obesity is an important source of metabolic and hormonal disruption, addressing these problems could have the potential to improve reproductive health outcomes and reduce the burden of obesity-related uterine disorders.
